# Rapid Detection of KPC-Producing Enterobacterales Susceptible to Imipenem/Relebactam by Using the MALDI-TOF MS MBT STAR-Carba IVD Assay

**DOI:** 10.3389/fmicb.2020.00328

**Published:** 2020-02-28

**Authors:** Marina Oviaño, Eva Gato, Germán Bou

**Affiliations:** Servicio de Microbiología, Complejo Hospitalario Universitario A Coruña, A Coruña, Spain

**Keywords:** MALDI-TOF, antimicrobial resistance, imipenem relebactam, carbapenemase resistance detection, clinical microbiology

## Abstract

*KPC*-producing Enterobacterales represent a serious public health concern. Limited therapeutic options are available for treatment, however, the novel combination of imipenem/relebactam represents a promising alternative. To preserve the activity of this new antibiotic combination, only targeted treatments will be recommended, and rapid tests to detect susceptible bacteria are therefore urgently needed. Here, we propose a MALDI-TOF-based method using the MBT STAR-Carba IVD assay, Bruker Daltonik, to detect KPC-producing Enterobacterales susceptible to imipenem/relebactam in a random selection of 143 clinical isolates previous molecular characterized, carrying 97 *bla*_KPC_, 1 *bla*_GES_, 12*bla*_VIM_, 4*bla*_IMP_, 3*bla*_NDM_, and 26*bla*_OXA–__48__–like_. Species identification was confirmed by MALDI-TOF MS. The molecular characterization of the isolates was performed by the Xpert Carba-R Assay and the results were used as gold standard. Besides, all isolates were submitted to imipenem and imipenem/relebactam microdilution susceptibility testing. The assay showed an overall sensitivity and specificity to detect class A-producing Enterobacterales susceptible to imipenem/relebactam of 98% (96/98) and 93% (42/45), respectively. This MALDI-TOF-based methodology, with a turnaround time of less than 1 h, is a reliable test for detecting imipenem/relebactam activity and its inclusion in routine laboratory screening would facilitate the correct use of this new combination of antimicrobials as a targeted treatment.

## Introduction

The global rise in the incidence of carbapenemase-producing Enterobacterales (CPE) is alarming and has posed a challenge to health services worldwide (Nordmann and Poirel, 2014). The increase in the prevalence of *Klebsiella pneumoniae* is especially worrying as these are the most frequent carbapenemase producers worldwide (Glasner et al., 2013). Limited therapeutic options are available for infections caused by CPE. Thus, only “second line” drugs such as polymyxins, tigecycline, aminoglycosides and fosfomycin may be active, although double carbapenem therapy can also be considered for use; finally, combination therapies are associated with better outcomes for high-risk patients (Rodríguez-Baño et al., 2018). Novel β-lactam inhibitors are being developed with the aim of restoring the activity of β-lactam antibiotics against CPE. Relebactam (MSD, EEUU) is an inhibitor of class A and C β-lactamases and is currently under clinical development in combination with imipenem-cilastatin. Imipenem/relebactam has recently undergone testing in phase 3 clinical trials for the treatment of patients with complicated intra-abdominal infections, complicated urinary tract infections and hospital-acquired/ventilator-associated bacterial pneumonia, with promising results. However, in order to reduce the spread of CPE and preserve the susceptibility to imipenem/relebactam, this treatment should only be used for infections that are proven or strongly suspected to be caused by susceptible bacteria to this antimicrobial combination. For new drugs, susceptibility testing is generally restricted to phenotypic tests like disc and/or gradient diffusion methods, until its inclusion in microdilution automated systems of susceptibility testing, all of which require at least 16 h to provide the results. MALDI-TOF MS has been used to detect carbapenems hydrolysis, as a surrogate test for susceptibility, and also to detect inhibition by β-lactam inhibitors (Oviaño and Bou, 2017, 2018; Carvalhaes et al., 2018; Anantharajah et al., 2019). EUCAST guidelines for carbapenemase detection also recommends the carbapenem hydrolysis assay by MALDI-TOF MS as a useful method for clinical practice. Here, we propose a rapid and simple MALDI-TOF MS-based assay for detecting class A-producing Enterobacterales, focused in KPC isolates due to their high prevalence, susceptible to imipenem/relebactam by using the commercial MBT STAR-Carba IVD assay.

## Materials and Methods

### Bacterial Isolates

The proposed method was applied in a random selection of 143 clinical isolates previous molecular characterized by using the Xpert Carba-R Assay (Cepheid, Sunnyvale, United States), carrying 97 *bla*_KPC_, 1 *bla*_GES_, 12*bla*_VIM_, 4*bla*_IMP_, 3*bla*_NDM_, and 26*bla*_OXA–__48__–like_ (Traczewski et al., 2018). The isolates were collected during a 2 months period (December 2017-January 2018) from 31 hospitals from Spain. The isolates were multidrug resistant. The molecular results were used as gold standard. Species identification was confirmed by MALDI-TOF MS. The carbapenemase-producing Enterobacterales (CPE) comprised 104 *Klebsiella pneumoniae*, 3 *K. oxytoca*, 12 *Escherichia coli*, 16 *Enterobacter cloacae*, 6 *Citrobacter freundii* and 2 *Serratia marcescens.* All isolates were submitted to imipenem and imipenem/relebactam microdilution susceptibility testing following the EUCAST guidelines. A decrease of at least three double serial dilutions in the imipenem/relebactam minimum inhibitory concentration (MIC) with respect to the imipenem MIC was considered for defining a positive inhibition. *E. coli* ATCC 25922 was used as a negative control, and a PCR-confirmed KPC-producing *E. coli* was used as a positive control.

### MALDI-TOF MS Assay

The proposed method was applied by using the MBT STAR-Carba IVD Kit in conjunction with the MBT STAR-BL IVD software (Bruker Daltonik, Germany). The test was performed according to the manufacturer’s instructions, with slight modifications regarding the inclusion of relebactam in the medium. After an overnight incubation at 37°C in Trypticase Soy Agar (Beckton Dickinson, Heidelberg, Germany), a 1 μl loop of bacteria was resuspended in a tube of lyophilized imipenem dissolved in 50 μl relebactam (3 mg/ml in MBT STAR Buffer). After incubation for 30 min at 37°C under agitation, the bacteria were pelleted by centrifugation (2 min at 14000 rpm), and 1 μl of the supernatant was spotted (in duplicate) onto a MALDI target. Air-dried spots were overlaid with MBT STAR Matrix.

### MALDI-TOF MS Analysis and Data Processing

The MBT STAR-Carba assay principle is the inactivation of imipenem by bacteria carrying carbapenemase enzymes due to the hydrolysis of the β-lactam ring. The hydrolysis reaction modifies the structure of the antibiotic that is associated with a mass shift that can be detected by MALDI-TOF. In cases of bacteria carrying carbapenemase enzymes inhibited by relebactam, no modification of imipenem could be observed. However, if the carbapenemase enzymes are not inhibited we could observe a proper hydrolysis of imipenem.

The mass spectrum was obtained using a MALDI Biotyper^®^ Smart (Bruker Daltonik) instrument, with Flex Control 3.4 software, in the m/z range of 100–1.000 Da. The MBT STAR-BL Software module was used to evaluate the spectra. The software automatically calculates the LogRQ value (which indicates the rate of hydrolysis) for imipenem in the presence of relebactam. According to the manufacturer’s instructions, normalized LogRQ values similar to or below 0.2 represent no proven imipenem hydrolysis. Normalized LogRQ values similar to or above 0.4 indicate proven imipenem hydrolysis. Normalized LogRQ values between 0.2 and 0.4 represent an ambiguous hydrolysis pattern which requires further testing or confirmation by other techniques.

## Results

**FIGURE 1:**
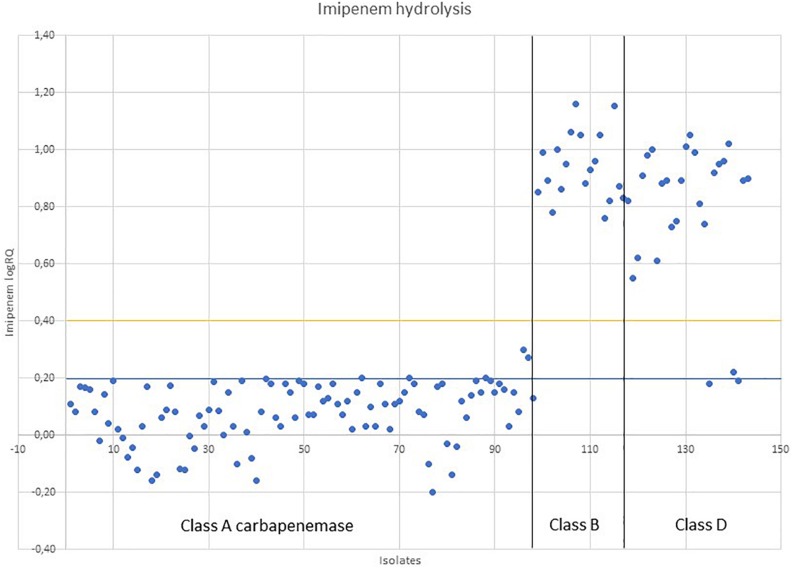
Imipenem/relebactam susceptibility indicated by MALDI-TOF MS, based on the measurement of imipenem hydrolysis in the presence of relebactam.

In this study, MALDI-TOF MS showed good results for detection of class A-producing Enterobacterales compared to the results of molecular methods, with a sensitivity of 98% (96/98) and a specificity of 93% (42/45) (Figure 1). The average logRQ for class A was 0.08 (*n* = 98), with two isolates proving an intermediate logRQ (0.30 and 0.27) (Supplementary Table S1). These isolates were retested as recommended by the manufacturer proving again an intermediate result. In this case, the MALDI-TOF MS could not provide a clear result of susceptibility and confirmation techniques should be further used. The average logRQ for class B (*n* = 19) was 0.94, with all isolates proving logRQ values above 0.4. The average logRQ for class D (*n* = 19) was 0.79, with all isolates proving logRQ values above 0.4, except one isolate that had an intermediate result (logRQ = 0.22) and two isolates that gave a negative result (logRQ = 0.18; 0.19). The isolate providing an intermediate result was also retested, providing the same value again.

Comparison with the results of imipenem/relebactam susceptibility testing was not possible as there are no clinical breakpoints established yet. Besides, as there has not been any correlation proven between the logRQ and the MIC in previous studies, we considered a criterion for relebactam inhibition an imipenem/relebactam MIC decrease in at least three double serial dilutions with respect to the imipenem MIC obtained by microdilution. Considering this criterion, we obtained a 100% sensitivity and specificity in detecting KPC-producing Enterobacterales susceptible to imipenem/relebactam (Supplementary Table S1), as all the isolates tested decreased in at least three double serial dilutions their MICs. For class B CPE the imipenem/relebactam MIC kept the same with respect to imipenem or in the minority of cases (2/19) decreased in one dilution. For class D CPE, the imipenem/relebactam MIC decreased in one dilution with respect to imipenem in the majority of isolates (21/26), whereas the minority of isolates (5/26) kept their MIC constant. There was no two or three double serial dilutions decrease observed for class B or D.

## Discussion

As there are few or no therapeutic alternatives available for treating infections caused by KPC-producing Enterobacterales, which are usually multidrug resistant, especially since the emergence of ceftazidime-avibactam resistance (Shields et al., 2017a, b) the early and accurate detection of imipenem/relebactam susceptibility in those isolates is extremely important in regard to prescribing this last resort antibiotic as a targeted treatment.

Our findings showed that the MALDI-TOF MS is an excellent tool for screening KPC-producing Enterobacterales susceptible to imipenem/relebactam before the microdilution results become available, providing results with a turnaround time of less than 1 h. Although, we only tested one GES-producing *K. oxytoca*, results seem consistent with those found in KPC-producing isolates. The sensitivity and specificity of the method is 98% (96/98) and 93% (42/45) respectively, compared to the molecular characterization of the isolates. When applying a second phenotypic criteria, as the decrease in at least three double serial dilutions of the imipenem/relebactam MIC with respect to the imipenem MIC obtained by microdilution, we obtained a 100% sensitivity and specificity. These results are in accordance to the imipenem/relebactam *in vitro* results found in previous studies that proved that the combination was active against class A CPE. Relebactam has not proved to be a good inhibitory substrate for class B and D CPE in previous studies (Lob et al., 2017; Karlowsky et al., 2018) as also found by MALDI-TOF MS. As expected, we did not detect relebactam inhibition in metallo-β-lactamase-producing isolates and in the majority of the class D CPE isolates. However, we recommend to perform the imipenem/relebactam MIC to assure the susceptibility of a carbapenem resistant Enterobacteral as it is considered the gold standard.

The isolate collection tested in this study was a reflection of our national CPE epidemiology and did not include any imipenem/relebactam resistant KPC-producing Enterobacteral which represents a limitation. Besides, the MALDI-TOF MS assay exclusively detects enzymatic carbapenem resistance but does not detect carbapenem resistance due to other mechanisms that can confer resistance as the OmpK35 disruption and/or mutated OmpK36 that have been described as chromosomal resistance in these isolates (Galani et al., 2019).

This method has only been applied to colonies in agar plates but could probably also be applied to positive blood cultures. The STAR MBT-Carba IVD assay is easy to use, and the handling time is very short. Moreover, the automated interpretation of results minimizes the inter-test variability associated with different environmental conditions and operators. Detailed analysis of spectra is not required, which simplifies the procedure and enables application of the assay by personnel who are not trained in mass-spectrometry techniques and its inclusion in routine in high throughput screening in microbiology laboratories.

In conclusion, this method is the first one to our knowledge to provide rapid results for an imipenem/relebactam early administration in KPC-producing Enterobacterales. Our results showed that the application of MALDI-TOF MS with the STAR MBT-Carba IVD assay, proved to be an excellent tool for screening imipenem/relebactam activity, helping in the antimicrobial therapy adjustment and early implementation of infection control measures.

Normalized imipenem LogRQ values for 143 CPE after 30 min of incubation with imipenem/relebactam following the MBT STAR-Carba IVD assay. LogRQ values above 0.4 (yellow line) mean positive imipenem/relebactam hydrolysis, values below 0.2 (blue line) mean negative imipenem/relebactam hydrolysis and intermediate values (>0.2 and <0.4) requires further testing.

## Data Availability Statement

All datasets generated for this study are included in the article/Supplementary Material.

## Author Contributions

MO and GB contributed to the conception and design of the study, and analyzed all the experiments. MO and EG performed the experiments. MO wrote the manuscript. GB contributed to the final version of the manuscript.

## Conflict of Interest

The authors declare that the research was conducted in the absence of any commercial or financial relationships that could be construed as a potential conflict of interest.
