# Supplementary material for: Rapid Detection of KPC-Producing Enterobacterales Susceptible to Imipenem/Relebactam by Using the MALDI-TOF MS MBT STAR-Carba IVD Assay
Source: Front Microbiol. 2020 Feb 28;11:328. doi: 10.3389/fmicb.2020.00328 (PMC7058919; doi:10.3389/fmicb.2020.00328)
Supplement: Supplementary file 1 [file Table_1.DOCX]

Supplementary Material

**Table 1.** Molecular characterization of the bacterial isolates used in the study, with the imipenem and the imipenem/relebactam MIC and the results of the imipenem hydrolysis assay by MALDI-TOF MS with the STAR-Carba IVD assay.

| **nº isolate** | **Isolate** | **resistance mechanism** | **Imipenem MIC** | **Imipenem/relebactam MIC** | **Inhibition by microdilution^1^** | **Imipenem/relebactam logRQ** | **MALDI-TOF MS hydrolysis of imipenem/relebactam^2^** |
| --- | --- | --- | --- | --- | --- | --- | --- |
| 1 | *C. freundii* | KPC | 4 | <=0,25 | + | 0,11 | - |
| 2 | *C. freundii* | KPC | 8 | <=0,25 | + | 0,08 | - |
| 3 | *K. pneumoniae* | KPC | 2 | 0,25 | + | 0,17 | - |
| 4 | *K. oxytoca* | KPC | 2 | <=0,12 | + | 0,17 | - |
| 5 | *K. pneumoniae* | KPC | 2 | <=0,25 | + | 0,16 | - |
| 6 | *K. pneumoniae* | KPC | 16 | <=0,25 | + | 0,08 | - |
| 7 | *K. pneumoniae* | KPC | 32 | <=0,25 | + | -0,02 | - |
| 8 | *K. pneumoniae* | KPC | 4 | <=0,25 | + | 0,14 | - |
| 9 | *K. pneumoniae* | KPC | 16 | <=0,25 | + | 0,04 | - |
| 10 | *K. pneumoniae* | KPC | 16 | <=0,25 | + | 0,19 | - |
| 11 | *K. pneumoniae* | KPC | 8 | <=0,25 | + | 0,02 | - |
| 12 | *K. pneumoniae* | KPC | 4 | <=0,12 | + | -0,01 | - |
| 13 | *K. pneumoniae* | KPC | 128 | 0,5 | + | -0,08 | - |
| 14 | *K. pneumoniae* | KPC | 4 | <=0,12 | + | -0,04 | - |
| 15 | *E. cloacae* | KPC | 2 | <=0,12 | + | -0,12 | - |
| 16 | *K. pneumoniae* | KPC | 64 | 0,25 | + | 0,03 | - |
| 17 | *K. pneumoniae* | KPC | 4 | <=0,12 | + | 0,17 | - |
| 18 | *K. pneumoniae* | KPC | 4 | <=0,12 | + | -0,16 | - |
| 19 | *S. marcescens* | KPC | 4 | <=0,12 | + | -0,14 | - |
| 20 | *E. cloacae* | KPC | 32 | 0,5 | + | 0,06 | - |
| 21 | *K. pneumoniae* | KPC | 4 | 0,5 | + | 0,09 | - |
| 22 | *K. pneumoniae* | KPC | 4 | <=0,12 | + | 0,17 | - |
| 23 | *K. pneumoniae* | KPC | 4 | <=0,12 | + | 0,08 | - |
| 24 | *K. pneumoniae* | KPC | 32 | <=0,12 | + | -0,12 | - |
| 25 | *E. cloacae* | KPC | 8 | 0,5 | + | -0,12 | - |
| 26 | *K. pneumoniae* | KPC | 2 | 0,25 | + | 0,00 | - |
| 27 | *K. pneumoniae* | KPC | 8 | 1 | + | -0,05 | - |
| 28 | *K. pneumoniae* | KPC | 16 | 1 | + | 0,07 | - |
| 29 | *K. pneumoniae* | KPC | 4 | 0,5 | + | 0,03 | - |
| 30 | *K. pneumoniae* | KPC | 8 | 1 | + | 0,09 | - |
| 31 | *K. pneumoniae* | KPC | 8 | 1 | + | 0,19 | - |
| 32 | *K. pneumoniae* | KPC | 8 | 0,5 | + | 0,08 | - |
| 33 | *K. pneumoniae* | KPC | 32 | <=0,25 | + | 0,00 | - |
| 34 | *K. pneumoniae* | KPC | 2 | <=0,25 | + | 0,15 | - |
| 35 | *K. pneumoniae* | KPC | 16 | <=0,12 | + | 0,03 | - |
| 36 | *K. pneumoniae* | KPC | 2 | 0,25 | + | -0,10 | - |
| 37 | *K. pneumoniae* | KPC | 8 | 0,5 | + | 0,19 | - |
| 38 | *K. pneumoniae* | KPC | 8 | <=0,12 | + | 0,01 | - |
| 39 | *K. pneumoniae* | KPC | 32 | <=0,12 | + | -0,08 | - |
| 40 | *K. pneumoniae* | KPC | 8 | <=0,12 | + | -0,16 | - |
| 41 | *K. pneumoniae* | KPC | 4 | <=0,12 | + | 0,08 | - |
| 42 | *K. pneumoniae* | KPC | 4 | 0,25 | + | 0,20 | - |
| 43 | *K. pneumoniae* | KPC | 16 | 1 | + | 0,18 | - |
| 44 | *K. pneumoniae* | KPC | 8 | 1 | + | 0,06 | - |
| 45 | *K. pneumoniae* | KPC | 32 | <=0,25 | + | 0,03 | - |
| 46 | *K. pneumoniae* | KPC | 8 | 0,5 | + | 0,18 | - |
| 47 | *K. pneumoniae* | KPC | 4 | 0,5 | + | 0,15 | - |
| 48 | *K. pneumoniae* | KPC | 16 | 0,5 | + | 0,06 | - |
| 49 | *K. pneumoniae* | KPC | 2 | <=0,12 | + | 0,19 | - |
| 50 | *K. pneumoniae* | KPC | 16 | 0,25 | + | 0,18 | - |
| 51 | *K. pneumoniae* | KPC | 4 | <=0,12 | + | 0,07 | - |
| 52 | *K. pneumoniae* | KPC | 64 | <=0,12 | + | 0,07 | - |
| 53 | *K. pneumoniae* | KPC | 8 | 0,5 | + | 0,17 | - |
| 54 | *K. pneumoniae* | KPC | 8 | <=0,12 | + | 0,12 | - |
| 55 | *K. pneumoniae* | KPC | 4 | <=0,12 | + | 0,13 | - |
| 56 | *K. pneumoniae* | KPC | 32 | <=0,12 | + | 0,18 | - |
| 57 | *K. pneumoniae* | KPC | 4 | 0,25 | + | 0,11 | - |
| 58 | *E. coli* | KPC | 8 | <=0,12 | + | 0,07 | - |
| 59 | *E. cloacae* | KPC | 64 | 1 | + | 0,12 | - |
| 60 | *K. pneumoniae* | KPC | 4 | 0,25 | + | 0,02 | - |
| 61 | *E. cloacae* | KPC | 8 | 0,5 | + | 0,15 | - |
| 62 | *K. pneumoniae* | KPC | 64 | 0,25 | + | 0,20 | - |
| 63 | *K. pneumoniae* | KPC | 64 | <=0,12 | + | 0,03 | - |
| 64 | *C. freundii* | KPC | 8 | <=0,12 | + | 0,10 | - |
| 65 | *K. pneumoniae* | KPC | 16 | <=0,12 | + | 0,03 | - |
| 66 | *E. cloacae* | KPC | 32 | 0,25 | + | 0,18 | - |
| 67 | *K. oxytoca* | KPC | 16 | 1 | + | 0,11 | - |
| 68 | *K. pneumoniae* | KPC | 2 | <=0,12 | + | 0,02 | - |
| 69 | *E. coli* | KPC | 4 | <=0,12 | + | 0,11 | - |
| 70 | *C. freundii* | KPC | 8 | <=0,12 | + | 0,12 | - |
| 71 | *K. pneumoniae* | KPC | 2 | <=0,12 | + | 0,15 | - |
| 72 | *K. pneumoniae* | KPC | 32 | <=0,12 | + | 0,20 | - |
| 73 | *K. pneumoniae* | KPC | 64 | <=0,12 | + | 0,18 | - |
| 74 | *E. coli* | KPC | 4 | <=0,12 | + | 0,08 | - |
| 75 | *E. cloacae* | KPC | 8 | 0,25 | + | 0,07 | - |
| 76 | *E. coli* | KPC | 4 | <=0,12 | + | -0,10 | - |
| 77 | *K. pneumoniae* | KPC | 4 | <=0,12 | + | -0,20 | - |
| 78 | *E. cloacae* | KPC | 32 | 0,5 | + | 0,17 | - |
| 79 | *C. freundii* | KPC | 4 | <=0,12 | + | 0,18 | - |
| 80 | *E. cloacae* | KPC | 8 | 0,5 | + | -0,03 | - |
| 81 | *K. pneumoniae* | KPC | 2 | <=0,12 | + | -0,14 | - |
| 82 | *C. freundii* | KPC | 1 | <=0,12 | + | -0,04 | - |
| 83 | *K. pneumoniae* | KPC | 4 | <=0,12 | + | 0,12 | - |
| 84 | *E. coli* | KPC | 1 | <=0,12 | + | 0,06 | - |
| 85 | *K. pneumoniae* | KPC | 8 | 0,25 | + | 0,14 | - |
| 86 | *K. pneumoniae* | KPC | 16 | 1 | + | 0,19 | - |
| 87 | *K. pneumoniae* | KPC | 32 | <=0,12 | + | 0,15 | - |
| 88 | *K. pneumoniae* | KPC | 16 | <=0,12 | + | 0,20 | - |
| 89 | *K. pneumoniae* | KPC | 32 | <=0,12 | + | 0,19 | - |
| 90 | *K. pneumoniae* | KPC | 32 | <=0,12 | + | 0,15 | - |
| 91 | *K. pneumoniae* | KPC | 8 | <=0,12 | + | 0,18 | - |
| 92 | *K. pneumoniae* | KPC | 32 | <=0,12 | + | 0,16 | - |
| 93 | *K. pneumoniae* | KPC | 64 | 0,5 | + | 0,03 | - |
| 94 | *K. pneumoniae* | KPC | 32 | <=0,12 | + | 0,15 | - |
| 95 | *K. pneumoniae* | KPC | 32 | <=0,12 | + | 0,08 | - |
| 96 | *K. pneumoniae* | KPC | 32 | <=0,12 | + | 0,30 | ± |
| 97 | *K. pneumoniae* | KPC | 16 | <=0,12 | + | 0,27 | ± |
| 98 | *K. oxytoca* | GES | 0,5 | <=0,12 | + | 0,13 | - |
| 99 | *E. cloacae* | VIM | 4 | 4 | - | 0,85 | + |
| 100 | *E. cloacae* | VIM | 2 | 2 | - | 0,99 | + |
| 101 | *E. cloacae* | VIM | 1 | 1 | - | 0,89 | + |
| 102 | *K. pneumoniae* | VIM | 2 | 2 | - | 0,78 | + |
| 103 | *E. cloacae* | VIM | 1 | 0,5 | - | 1,00 | + |
| 104 | *E. cloacae* | VIM | 0,5 | 0,5 | - | 0,86 | + |
| 105 | *E. coli* | VIM | 1 | 1 | - | 0,95 | + |
| 106 | *K. pneumoniae* | VIM | 1 | 1 | - | 1,06 | + |
| 107 | *K. pneumoniae* | VIM | 4 | 4 | - | 1,16 | + |
| 108 | *K. pneumoniae* | VIM | 32 | 32 | - | 1,05 | + |
| 109 | *E. cloacae* | VIM | 2 | 1 | - | 0,88 | + |
| 110 | *E. cloacae* | VIM | 256 | 256 | - | 0,93 | + |
| 111 | *E. coli* | IMP | 2 | 2 | - | 0,96 | + |
| 112 | *K. pneumoniae* | IMP | 1 | 1 | - | 1,05 | + |
| 113 | *K. pneumoniae* | IMP | 0,5 | 0,5 | - | 0,76 | + |
| 114 | *K. pneumoniae* | IMP | 0,5 | 0,5 | - | 0,82 | + |
| 115 | *K. pneumoniae* | NDM | 8 | 8 | - | 1,15 | + |
| 116 | *E. coli* | NDM | 8 | 8 | - | 0,87 | + |
| 117 | *E. coli* | NDM | 16 | 16 | - | 0,83 | + |
| 118 | *E. coli* | OXA-48 | 2 | 2 | - | 0,82 | + |
| 119 | *K. pneumoniae* | OXA-48 | 4 | 4 | - | 0,55 | + |
| 120 | *K. pneumoniae* | OXA-48 | 8 | 4 | - | 0,62 | + |
| 121 | *K. pneumoniae* | OXA-48 | 1 | 1 | - | 0,91 | + |
| 122 | *K. pneumoniae* | OXA-48 | 4 | 2 | - | 0,98 | + |
| 123 | *E. coli* | OXA-48 | 64 | 32 | - | 1,00 | + |
| 124 | *E. coli* | OXA-48 | 1 | 0,5 | - | 0,61 | + |
| 125 | *K. pneumoniae* | OXA-48 | 2 | 1 | - | 0,88 | + |
| 126 | *K. pneumoniae* | OXA-48 | 16 | 8 | - | 0,89 | + |
| 127 | *K. pneumoniae* | OXA-48 | 4 | 2 | - | 0,73 | + |
| 128 | *S. marcescens* | OXA-48 | 8 | 4 | - | 0,75 | + |
| 129 | *K.pneumoniae* | OXA-48 | 0,5 | 0,25 | - | 0,89 | + |
| 130 | *K.pneumoniae* | OXA-48 | 0,5 | 0,5 | - | 1,01 | + |
| 131 | *K.pneumoniae* | OXA-48 | 1 | 0,5 | - | 1,05 | + |
| 132 | *K.pneumoniae* | OXA-48 | 0,25 | <=0,12 | - | 0,99 | + |
| 133 | *K.pneumoniae* | OXA-48 | 0,25 | <=0,12 | - | 0,81 | + |
| 134 | *K.pneumoniae* | OXA-48 | 64 | 32 | - | 0,74 | + |
| 135 | *K.pneumoniae* | OXA-48 | 0,25 | <=0,12 | - | 0,18 | - |
| 136 | *K.pneumoniae* | OXA-48 | 0,25 | <=0,12 | - | 0,92 | + |
| 137 | *K.pneumoniae* | OXA-48 | 2 | 2 | - | 0,95 | + |
| 138 | *K.pneumoniae* | OXA-48 | 1 | 0,5 | - | 0,96 | + |
| 139 | *K.pneumoniae* | OXA-48 | 2 | 1 | - | 1,02 | + |
| 140 | *K.pneumoniae* | OXA-48 | 2 | 1 | - | 0,22 | ± |
| 141 | *K.pneumoniae* | OXA-48 | 1 | 0,5 | - | 0,19 | - |
| 142 | *K.pneumoniae* | OXA-48 | 0,5 | 0,25 | - | 0,89 | + |
| 143 | *K.pneumoniae* | OXA-48 | 0,5 | 0,25 | - | 0,90 | + |

^1^ The criteria considered for relebactam inhibition was a decrease in at least three double serial dilutions of the imipenem/relebactam MIC with respect to the imipenem MIC obtained by microdilution.

^2^ A negative hydrolysis of imipenem/relebactam, is represented by (-), an intermediate hydrolysis is represented by (±) and a positive hydrolysis is represented by (+).
